
JOURNAL INFORMATION
==============================
NLM Title Abbreviation: ScientificWorldJournal
Journal ID: ScientificWorldJournal
NLM Journal ID: 101131163
Journal ID: TSWJ

The Scientific World Journal

ISSN: 2356-6140
EISSN: 1537-744X
Publisher: Wiley

ARTICLE INFORMATION
==============================
PMCID: PMC5936531
DOI: 10.1100/tsw.2005.61
Article ID: 798718
Article version: 1
Subjects: Editorial

Hematology-Aims and Scope and a Call for Papers

Benz Edward *
Harvard University, Dana Farber Cancer Institute, Boston, MA, USA
*Edward Benz: edward_benz@dfci.harvard.edu
Publication date: 2005
Electronic publication date: 2005 Jun 8
Volume: 5
First page: 469
Last page: 469
Copyright: Copyright © 2005 Edward Benz.
Copyright year: 2005
License: This is an open access article distributed under the Creative Commons Attribution License, which permits unrestricted use, distribution, and reproduction in any medium, provided the original work is properly cited.
License URL: https://creativecommons.org/licenses/by/3.0/

==============================
Editorial
*TheScientificWorldJOURNAL* (2005) 5, 469
ISSN 1537-744X; DOI 10.1100/tsw.2005.61

### Hematology - Aims and Scope and a Call for Papers

Edward Benz
*Harvard University, Dana Farber Cancer Institute, Boston, MA*

E-mail: edward_benz@dfci.harvard.edu

*Published June 8, 2005*

The Hematology Domain of TheScientificWorldJOURNAL aims to provide the learner with authoritative information, provided by international experts, in the broad areas of hematology and oncology. The emphasis will be on the relationships between state of the art basic science discoveries, potential clinical applications, and active translational research that promises to apply these discoveries to improved modalities of detection, diagnosis, and treatment.

Hematology and oncology are clinical disciplines that are particularly driven by basic science research, given the nature of the diseases that fall within the scope of these specialties. Diseases of the blood and neoplastic diseases are fundamentally cellular processes caused by disruptions, usually at the genomic level, or by altered immunologic response, which in turn is often driven at the genomic level. Study of the normal physiology of the relevant cells, and of the derangements responsible for neoplastic disorders and disorders of the blood have thus benefited enormously from the human genome project and the vastly improved technological platforms that have evolved from it.

The Hematology Domain welcomes reviews covering the basic cellular and chemical components of the blood (red cells, white cells, platelets, clotting proteins, etc.) and the molecular pathophysiology of major blood disorders, such as hemoglobinopathies, bone marrow failure syndromes, coagulopathies, etc., neoplasms of the blood cells and blood forming organs, e.g., leukemia, lymphoma, myeloproliferative and myelodysplastic syndromes, immune deficiencies, etc. Finally, aspects of oncology that are being impacted by advances in basic science, e.g., targeted therapies, and angiogenesis, etc., will follow within the scope of this initiative.

In each case, authors will be instructed to prepare a clear exposition of the scientific advances readable by well-informed but not necessarily expert professionals, and to delineate how those scientific advances promise to impact the detection, diagnosis, and treatment of the disorders. Whenever possible, topics will be chosen in areas in which actual progress is being made in translating new scientific information into altered clinical management of patients.

**This article should be referenced as follows:**

Benz, Edward. (2005) Hematology - Aims and Scope and a Call for Papers. *TheScientificWorldJOURNAL* **5**, 469.

*Corresponding author.
©2005 with author.
